# The Adventures of the "Berry Mission."

**Published:** 1916-10-07

**Authors:** 


### A RED CROSS UNIT IN SERBIA.

### The Adventures of the "Berry Mission."

As is foreshadowed on the last page of the narrative of the work and experiences in Serbia of the Red Cross unit taken out there at the beginning of 1915 by Mr. and Mrs. James Berry,* those two philo-Slavonic enthusiasts have now transferred their services and energies to the cause of the Russian wounded. It is possible, but perhaps not likely, that their new sphere of action may provide them with adventures as interesting and problems as arduous as those encountered in Serbia.

### A Many-sided Specialist.

Besides being a hospital surgeon of very senior standing and ripe experience, Mr. Berry is distinctly one of the personalities of the London surgical world; and in some ways this book discloses all too little of his work as head of the unit upon which his name was very generally, though unofficially, bestowed. In the chapters written by himself he represses all self-revelation and in the chapters written by his collaborators there is but a little light thrown on his work as head of the unit. Of his energies as sanitary reformer, architect, swamp-drainer, and (occasionally) as surgeon we hear a great deal. But of his acts and principles as organiser, commander, disciplinarian, there is tantalisingly small account, though sundry hints are dropped about occasions of mild discontent, which sometimes became vocal, on the part of various members of the mission. It is true that Mr. Berry in his concluding chapter indicates some of the problems which heads of voluntary (and mainly unpaid) Red Cross units should be able to solve; but he does not tell us how he himself met these difficulties. However, it is fairly clear that his tact, his example, and his indomitable energy kept the unit remarkably well together in very trying times; and he is probably correct in his claim that it had less friction than any other in Serbia. It is notable however, that a good many of his workers left for home after comparatively short periods of service.

### Sanitation in Serbia.

From one circumstance and another over which the members of the mission had no control, there was remarkably little surgical work done during the year of the unit's sojourn in Serbia. What professional work was accomplished seems to have been largely medical; but even the typhus epidemic was over in two or three months after the unit got to work. It need not be inferred, however, that little was effected; on the contrary, the enthusiasm which Mr. Berry and others threw into the task of introducing the elements of hygiene to the notice of the Serbian people was pioneer work of a particularly valuable kind in so primitive a country, and will in all likelihood be remembered there long after the rest of their labours have been forgotten. This attention to sanitation and health questions need not alarm prospective lay readers; the topics are so handled as not to repel the sensibilities of any but those delicately minded mortals to whom the very names of domestic vermin are completely *tabu*.

Of even greater general interest are the impressions of the authors on the qualities of the Serbs, Czechs, Magyars, Germans, Rumanians, and other races with whom as patients or as prisoner-orderlies the mission had to deal. These topics occupy, indeed, the greater part of the whole book, and are handled with refreshing sympathy, humour, and penetration. Naturally the Serbians receive most attention; of these "Irish of the Balkans" all the contributors speak in terms of affection, as do most other authorities who have dwelt with them long enough to get below the surface of their national peculiarities. In the wards, it is shown, complete amity and fraternity existed between all the many races commingled there, even including the Austrians of German nationality. The pictures of mutual toleration and intimacy among all these different races in the middle of a disastrous war are wholly delightful and human. The Austrian prisoners needed no guards, for they were not possessed by the very slightest anxiety to return across the Danube to fight in the Austrian army; and apparently many of them fled before the invading columns with as great an anxiety to avoid falling into the hands of their friends as the unhappy Serbians felt to escape from the treacherous Bulgars or their Austro-German allies. Altogether, in spite of the lack of homogeneity due to diverse authorship, this attractive book is well worth reading by anyone who is interested in either Serbia or in Red Cross work in strange lands. It exhibits at their best the English qualities of adaptability, good sense, energy, and generosity; and it is impossible to read it without realising that the official tributes of the Serbian Crown Prince and other authorities to the work of the Berry Mission were both sincere and well deserved.

* *A Red Cross Unit in Serbia.* By James Berry, F.R.C.S.; Mrs. F. M. D. Berry, M.D.; W. L. Blease, L.L.M.; and others. London: J. and A. Churchill, 1916. Pp. 293. Price 6s. net.

